# Usefulness of Noncontrast MRI-Based Radiomics Combined Clinic Biomarkers in Stratification of Liver Fibrosis

**DOI:** 10.1155/2022/2249447

**Published:** 2022-06-21

**Authors:** Ru Zhao, Hong Zhao, Ya-Qiong Ge, Fang-Fang Zhou, Long-Sheng Wang, Hong-Zhen Yu, Xi-Jun Gong

**Affiliations:** ^1^Department of Radiology, The Second Affiliated Hospital of Anhui Medical University, 678 Furong Road, Hefei 230601, Anhui, China; ^2^GE Healthcare China, Pudong New Town, No. 1, Huatuo Road, Shanghai 210000, China; ^3^Department of Pathology, The Second Affiliated Hospital of Anhui Medical University, 678 Furong Road, Hefei 230601, Anhui, China

## Abstract

**Purpose:**

To develop and validate a radiomic nomogram based on texture features from out-of-phase T1W images and clinical biomarkers in prediction of liver fibrosis.

**Materials and Methods:**

Patients clinically diagnosed with chronic liver fibrosis who underwent liver biopsy and noncontrast MRI were enrolled. All patients were assigned to the nonsignificant fibrosis group with fibrosis stage <2 and the significant fibrosis group with stage ≥2. Texture parameters were extracted from out-of-phase T1-weighted (T1W) images and calculated using the Artificial Intelligent Kit (AK). Boruta and LASSO regressions were used for feature selection and a multivariable logistic regression was used for construction of a combinational model integrating radiomics and clinical biomarkers. The performance of the models was assessed by using the receiver operator curve (ROC) and decision curve.

**Results:**

ROC analysis of the radiomics model that included the most discriminative features showed AUCs of the training and test groups were 0.80 and 0.78. A combinational model integrating RADscore and fibrosis 4 index was established. ROC analysis of the training and test groups showed good to excellent performance with AUC of 0.93 and 0.86. Decision curves showed the combinational model added more net benefit than radiomic and clinical models alone.

**Conclusions:**

The study presents a combinational model that incorporates RADscore and clinical biomarkers, which is promising in classification of liver fibrosis.

## 1. Introduction

Liver disease accounts for approximately 2 million deaths per year and is the main public health crisis worldwide [1]. Liver fibrosis is the main procedure for hepatitis processing to cirrhosis and is characterized by the deposition of extracellular matrix in the liver. Cirrhosis is currently the 11^th^ most common cause of death globally and may be accompanied by several complications (i.e., hepatocellular carcinoma and portal hypertension) which may relate to disability and life loss. Promising studies have shown that fibrosis can be eliminated or reversed in the early stage, while cirrhosis is difficult to be reversed [2, 3]. So, early diagnosis of liver fibrosis is rather important in liver disease.

Although biopsy is still the golden standard for liver fibrosis staging, it has some limitations, for instance, the invasive way, poor repeatability, sampling error, and hospitalization [4, 5]. A noninvasive surrogate method for liver fibrosis assessment is under development. The aspartate transaminase-to-platelet ratio index (APRI) and the fibrosis 4 index (FIB-4) are widely used clinical biomarkers in staging liver fibrosis for their easy accessibility [6]. But APRI and FIB-4 were constructed based on hepatitis C patients and had better performance in differentiating advanced fibrosis and cirrhosis [6]. Transient elastography (TE) and MR elastography (MRE) are the available imaging tools for predicting liver fibrosis [7]. But TE is vulnerable to obese and ascites, while MRE is in a high requirement of hardware [8].

Radiomics analysis is a promising method in predicting liver fibrosis according to large amounts of studies. Texture features extracted from contrast MR images, T1 and T2 mapping images, and diffusion-weighted images all exhibited good to excellent diagnostic values [9–13]. Previous studies have shown that texture features based on T2W and T1W images have the potential to stratify liver fibrosis, while few studies have explored the usefulness of texture features from out-of-phase T1WI in the classification of fibrosis [9, 14, 15]. Though our previous study had shown that texture analysis based on in-phase and out-of-phase T1W and T2W images had good diagnostic accuracy in differentiating early stage fibrosis (F0-F1) and significant fibrosis (F2–F4) in chronic hepatitis B infection patients, the texture model from out-of-phase T1W images had a higher AUC than that from in-phase T1W and T2W images [16]. Thinking about the limited number of patients, the value of texture features from out-of-phase T1WI in classification of liver fibrosis still needs to be discussed. According to the European Association for the Study of the Liver (EASL) guidelines for hepatitis B, hepatitis C, and nonalcoholic fatty liver disease [17–19], chronic hepatitis patients with fibrosis stage >2 or higher should accept drug treatment. In this retrospective study, we will investigate the usefulness of a combination of radiomics signatures from out-of-phase T1W images and clinical biomarkers of chronic liver disease in differentiating significant and nonsignificant liver fibrosis.

## 2. Materials and Methods

This retrospective study was approved by the institutional review board and local ethics committee (no. PJ2016-013-01). Written informed consent was obtained. All liver biopsies were clinically indicated.

### 2.1. Study Participants

Participants who were clinically diagnosed with chronic hepatitis and underwent both noncontrast MRI scan and liver biopsy were screened for this study between July 2016 and September 2021. Inclusion criteria were as follows: adult patients 18 years of age or older, patients who underwent MRI scans before undergoing liver biopsies, patients' timeframe between MRI and biopsy should be six months, and patients who are willing to participate in this study and signed a written informed consent form. Exclusion criteria were as follows: patients with claustrophobia, MR images with large respiration or motion artifacts, decompensated cirrhosis, patients with splenectomy, and MRI exams performed 6 months or later than biopsy. A total of 145 participants were screened and 139 were included.

### 2.2. MRI Data Acquisition of the Liver

All MRI scans were performed on the same 1.5 T clinical system (Avanto, Siemens Healthcare, Erlangen, Germany) using a 4-channel body phased-array coil. All participants underwent the same abdominal MRI protocol, which consisted of the following sequences: in-phase and out-of-phase T1-weighted axial images. The imaging parameters of the T1W sequence were TR (repetition time) 200 ms, TE (time to echo) 2.2 ms/4.4 ms (in-phase/out-of-phase), averages of 1, concatenations 1, FoV (field of view) read 380 mm, FoV phase 78.1%, and slice thickness 6.0 mm.

### 2.3. Histological Analysis

A liver biopsy was performed under ultrasound guidance. Histopathologic features were evaluated by a pathologist with 10 years of experience who was blinded to patients' MRI diagnoses. The biopsy sampling area was selected in the liver at the level of the midaxillary line of the 8^th^ rib. The fibrosis stages were assessed according to the METAVIR scoring system [20] and standardized to the common scale. Standardization was as follows: F0, no fibrosis; F1, portal fibrosis without septa; F2, portal fibrosis with rare septa; F3, numerous septa without cirrhosis; and F4, cirrhosis.

### 2.4. Clinical Biomarkers

Laboratory results one week before biopsy were collected from the electronic medical record. Biomarkers included alanine aminotransferase (ALT), aspartate aminotransferase (AST), and platelet count (PLT). APRI was calculated using the following formula: (AST/upper normal limit × 100/platelet counts), and FIB-4 was calculated using the following formula: (age × AST)/(platelet counts × ALT^1/2^).

### 2.5. Acquisition of Texture Features

**Figure 1:**
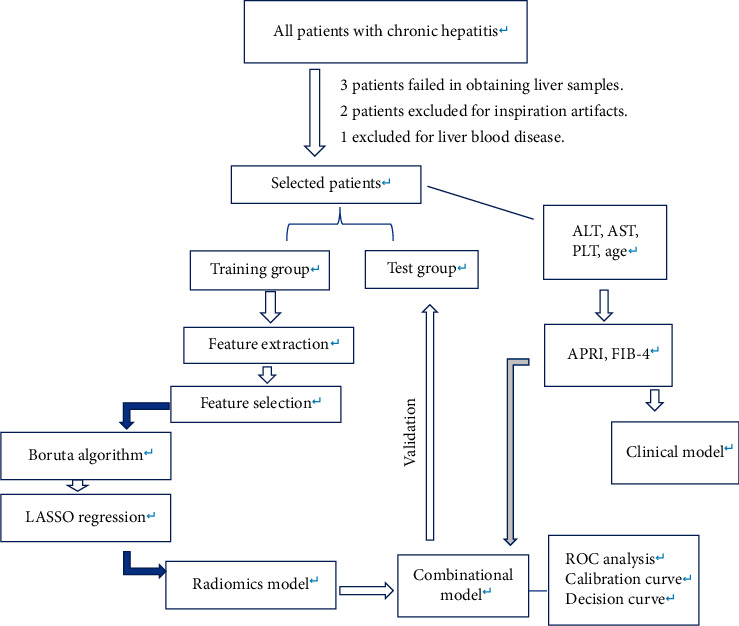
Flowchart of feature selection, model construction, and validation.

Texture features were extracted from out-of-phase T1W images of all participants by two radiologists (with 8 and 5 years of experience in abdominal imaging diagnosis, respectively). All participants' images were exported in DICOMS format and then imported into an open-source software program (ITK-SNAP, V3.30) [21] for manual receiver of interest (ROI) delineation. For each sequence, two continuous slices of the right lobe [22] with no liver lesions and a low number of motion artifacts were selected. Two free-hand ROIs of the continuous slices, as large as possible and avoiding major blood vessels or liver lesions, were placed on out-phase T1WI and laid out as an ROI image. All images were processed by using the AK software (Artificial Intelligence Kit Version; V3.2.0R, GE Healthcare, Shanghai), which complied with IBIS (image biomarker standardization initiative). Images were resampled with dimensions of 1 *∗* 1 *∗* 1 mm by the linear interpolation method, and image intensity was normalized with Z-core standardization (mean of 0 and deviation of 1). Radiomics features include the first order, shape, gray-level co-occurrence matrix, gray-level run-length matrix, neighboring gray one-tone difference matrix, gray-level differential matrix, and wavelet transform. Overall, 851 texture features were extracted per ROI. Details are shown in Figure 1.

### 2.6. The Intraobserver and Interobserver Agreements

The intraclass correlation coefficient (ICC) [23, 24] was applied to analyze the intraobserver and interobserver agreements of the feature extraction. Thirty randomly selected patients' data were used for ICC analysis. Reader A delineated the ROIs twice on two different weeks to evaluate the intraobserver agreement and reader B independently delineated them once to evaluate the interobserver agreement with the ROIs delineated by reader A. Features with mean values of the intraclass and interclass ICC higher than 0.75 were retained.

## 3. Statistical Analysis

All participants were randomly assigned into training group and test group with a ratio of 7 : 3. Considering the imbalance of the data, SMOTE was used for data augmentation of the minority group by increasing synthetic data points based on the original data points. All statistic data were performed by using R 3.5.1R (The R Project for Statistical Computing (r-project.org)) software. In the training group, two feature selection methods, Boruta and Least absolute shrinkage and selection operator (LASSO) [25], were used to select features. Boruta [26] is an all-relevant feature selection wrapper built around the random forest classification algorithm, capable of working with any classification method that outputs a variable importance measure (VIM). The method performs a top-down search for relevant features by comparing original attributes' importance with the importance achievable at random, estimated using their permuted copies, and progressively eliminating irrelevant features to stabilize that test. Then, LASSO was conducted to choose the optimized subset of features and multivariable logistic regression was used to construct the radiomics model. RADscore of each patient was calculated by summing the selected features weighted by their regression coefficients. The discriminative ability of the RADscore was compared by using the Wilcoxon test and receiver operating characteristic curve (ROC) analysis.

For clinical factors, continuous variables were analyzed by using the independent *t*-test or Wilcoxon test. Categorical variables were analyzed by the chi-square test or Fisher's test. Univariable logistic regression was used to evaluate the association between clinical factors and the fibrosis grades. Finally, a clinical model was built by multivariable logistic regression. Meanwhile, collinearity was calculated based on the variance inflation factor (VIF) and a feature with VIF >10 was removed, and validation was applied by ROC analysis in the training and test groups.

Significant clinical factors, as well as radiomics scores, were analyzed in multivariable regression to construct the combination model. The discriminative performance of the model was assessed using the ROC analysis and calibration curve. The Hosmer–Lemeshow test was used to test the goodness-of-fit of the model both in the training and test groups. The Delong test was applied to test the area under the ROC curve (AUC) of different models. A decision curve (DCA) was implemented to verify the clinical usefulness of the combination model, clinical model, and radiomic model, by calculating the standardization net benefits at different threshold probabilities. *P* < 0.05 was considered statistical significance.

## 4. Result

A total of 139 patients (female 64, male 75) were enrolled in this study, with 42 with fibrosis stage 1 (*F* = 1), 33 with stage 2 (*F* = 2), 32 with stage 3 (*F* = 3), and 32 with cirrhosis. The underlying diagnoses for liver biopsy were hepatitis B infection in 82 patients, hepatitis C infection in 4 patients, 15 patients with autoimmune hepatitis, 10 primary biliary hepatitis, 10 drug-induced hepatitis, and 18 others (including overlap syndrome and alcoholic hepatitis). All patients were assigned into nonsignificant fibrosis (*F* < 2) (age 47.5 ± 3.53) and significant fibrosis (*F* ≥ 2) (age 49.27 ± 19.79). Then patients' omics data were randomly divided into training and test groups with a ratio of 7 : 3.

### 4.1. Interobserver and Intraobserver Agreement

The ICC of the interobserver of the two independent readers ranged from −0.39 to 0.99, and the ICC of the intraobserver of the same reader ranged from −0.75 to 0.99. A total of 176 features were retained and all showed high interobserverand intraobserver ICCs with ICCs ≥  0.75. Thus, features extracted by reader A were used for further analysis.

### 4.2. Radiomics Model Construction

**Figure 2:**
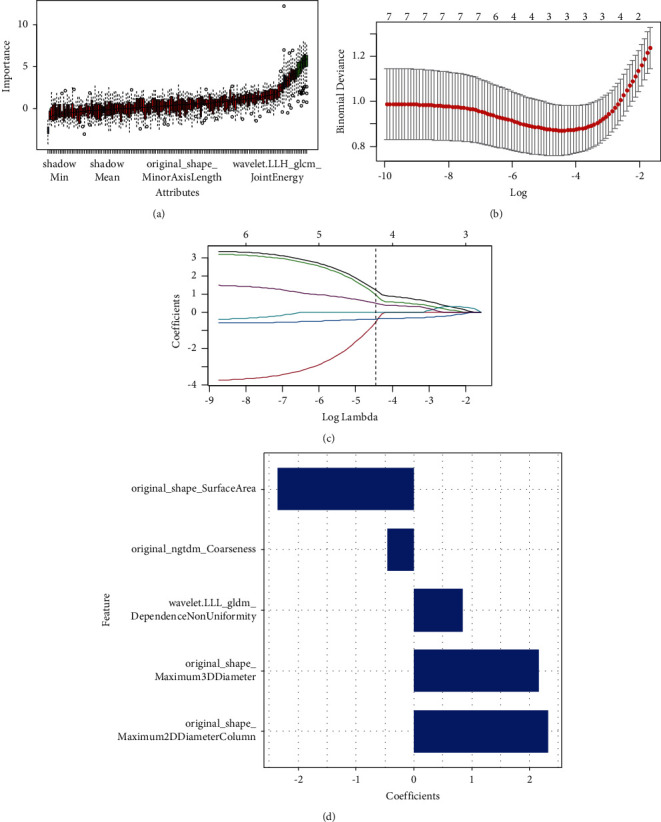
Result of feature selection of boruta and least absolute shrinkage and selection operator (LASSO) for significant fibrosis (*F* ≥ 2). (a) Importance and attributes of significant fibrosis (*F* ≥ 2) in boruta. Red represents the rejected attribute and green is the confirmed attribute. Blue represents uncertain attributes. (b), (c) The bottom *X*-axis represents the value of log (*λ*), while the upper *X*-axis represents the number of nonzero parameters. A dotted vertical line was drawn at the optimal values by using the minimum criteria with (*λ*) 0.00416. (d) Coefficient of the most predictive features.

**Figure 3:**
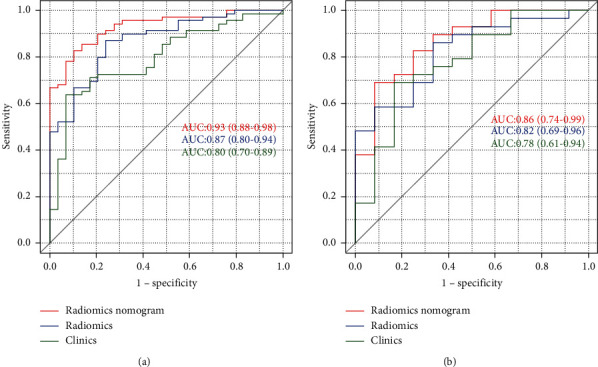
The area under receiver operator curve (AUROC) of the training group (a) and test group (b).

After the Boruta and LASSO regression processes, the five most predictive features were retained as shown in Figure 2. Then, a radiomic model was conducted and RADScores were all calculated. The Wilcoxon test exhibited that discrimination of RADscore had a statistical difference both in training and test sets. ROC analysis showed that AUCs of the training and test groups were 0.87 (95% CI: 0.80–0.94) and 0.82 (95% CI: 0.69–0.96). Details are shown in Figure 3.

### 4.3. Clinical Model Construction

Results of univariable logistic regression of the clinical factors are FIB-4 2.03 (95% CI: 1.43–3.29) (*P*=0.001) and APRI 1.8 (95% CI: 1.16–3.40) (*P*=0.032). Then, the clinical model was constructed, and ROC analysis showed that the AUCs of the training and test groups were 0.80 (95% CI: 10.70–0.89) and 0.78 (95% CI: 10.61–0.94). Details are showned in Figure 3.

### 4.4. Combinational Model Construction

**Figure 4:**
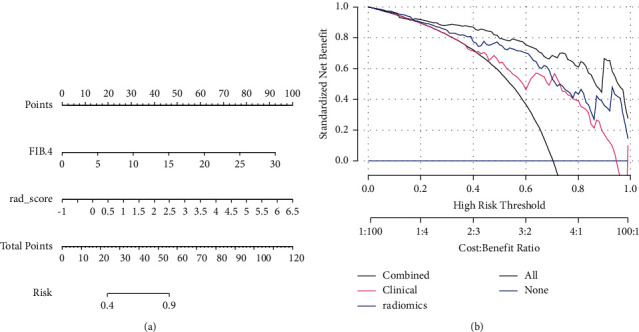
Nomogram (a) and decision curve (b). Decision curve showed more benefit than the treat-all (slash line) and treat-none (horizontal line) schemes and the combined model added more benefits than the radiomics and clinical model.

**Figure 5:**
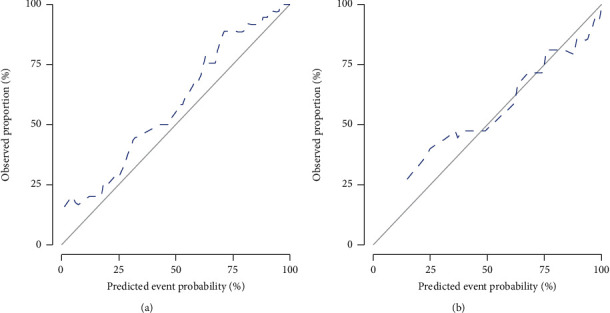
Calibration curve ((a) training group and (b) test group). Calibration curves exhibited excellent good of fit.

After the multivariable logistic regression, RADscore and FIB-4 were included in the final combinational model, while APRI was excluded for collinearity. The AUCs of the training and test groups of the combinational model were 0.93 (95% CI: 0.88–0.98) and 0.86 (95% CI: 0.74–0.99). The Delong test of AUC showed AUC of the combinational model and clinical model had statistical significance, while the others showed no statistical significance. The calibration curve and Hosmer–Lemeshow test exhibited acceptable goodness of fit with *P* > 0.05. The decision curve of the radiomic model, clinical model, and combinational model all yielded more benefits than the treat-all and treat-none schemes and indicated that the combinational model provided much more net benefit than the radiomic model and clinical model. Details are shown in Figures 3–5 and Table 1.

## 5. Discussion

In this study, we developed and validated a noncontrast MRI radiomics nomogram for classification of liver fibrosis which incorporated the most predictive radiomics signatures and FIB-4 and had the potential to classify nonsignificant and significant fibrosis in a noninvasive way. Liver fibrosis is an important dynamic course during hepatitis processing to cirrhosis and is caused by the deposition of extracellular matrix. Liver fibrosis is difficult to diagnose because fibrosis is characterized by subtle changes and cannot be visible using CT or MRI examinations. Texture analysis is a new method that can extract features from images and transform them into visible variables. By analyzing these variables, more subtle changes inside the images can be obtained for further study.

For the construction of the radiomics model, the Boruta algorithm and LASSO regression were used for feature selection because of the redundancy of omics features. Boruta was developed to identify all relevant variables in a more efficient and stable way. It has been proved that Boruta was the most powerful approach for high-dimensional omics datasets in machine learning. LASSO regression is characterized by the simultaneous fitting of generalized linear models and variable selection based on the strength of their univariable outcomes and regularization. The AUC of the radiomics model of the training group is 0.87 (0.80–0.94) and validated in the test group with an AUC of 0.82 (0.69–0.96). It revealed a radiomics model on the basis of out-of-phase MRI had the potential for classification of liver fibrosis. Large numbers of previous studies have proven that radiomics analysis of CT and MRI images could be a surrogate method for predicting liver fibrosis [10, 13, 27–29]. Jia et al. [30] extracted histogram and texture parameters of T1 maps of low and high risk of advanced fibrosis and constructed a multivariate model integrating median, 5^th^ percentile, and diff-entropy and reported an AUC of 0.902 in stratification of advanced fibrosis. Park et al. [31] calculated the radiomics fibrosis index (RFI) by using a radiomics model based on gadoxetic acid-enhanced hepatobiliary phase MRI between stages F0–F2 and F3-F4 and found that RFI significantly outperformed normalized liver enhancement, APRI, and FIB-4 for staging fibrosis. Schawkat et al. [9] compared the diagnostic accuracy of texture analysis combined with machine learning of noncontrast-enhanced T1W and T2W images and depicted a similar AUC compared to MRE for texture analysis from T1W images and a significantly lower AUC of T2W images compared to MRE for classification of lower-stage fibrosis and high-stage fibrosis. Radiomics based on noncontrast MRI scans is promising in assessment of liver fibrosis and may have potential help in evaluation of fibrosis in antiviral therapy patients. Direct-acting antiviral (DAA) therapy is popular in hepatitis C virus treatment which can dramatically improve sustained virological response [32]. Radiomics combined with MRI would be relevant to detecting the fibrosis stage and establishing a priority of DAA treatment for hepatitis C patients with fibrosis stage *F* ≥ 2.

APRI and FIB-4 are the most widely used clinical biomarkers for classification of liver fibrosis. Hu et al. [33] reported that the AUC of the training cohort and test cohort of a clinical model integrating FIB-4 and APRI were 0.731 and 0.703 for differentiating advanced fibrosis and cirrhosis, while in our study, the AUC of the clinical model in the training and test groups were 0.80 and 0.78 and showed poor performance. Hu et al. [33] constructed a nomogram based on portal venous phase CT images and reported a diagnostic accuracy of 0.778 and 0.778 in the training and test cohorts with an AUC of 0.864 and 0.772 for differentiating nonadvanced from advanced fibrosis. In this study, a combinational model incorporated with RADScore and clinic biomarkers was constructed, and ROC analysis exhibited good to excellent performance in the training and test groups, with diagnostic accuracy of 0.85 and 0.76 and an AUC of 0.93 and 0.86. The decision curve indicated more net benefit than the radiomics and clinical models alone. The nomogram of the combinational model made the prediction of significant liver fibrosis more accessible and visible. It revealed that a combinational model of RADscore and clinical biomarkers is promising in differentiating nonsignificant and significant liver fibrosis. However, APRI and FIB-4 entailed a risk of overestimating the fibrosis stage due to the impact of the necroinflammatory activity on transaminases [34]. More stable serological markers incorporated with RADscore should be discovered. Recently, Mohamed et al. [35] found that serum levels of vitronectin increased significantly in cirrhosis patients than in controls. It may reveal that a combination of RADscore and vitronectin may be promising in classification of liver fibrosis.

There are also some limitations in our study. First, the limited sample size in this study might decrease the statistical power and external validation should be required in another cohort. Second, the lack of fibrosis stage F0 may add some biases to the results. Third, considering the limited number of patients, we included all patients with different kinds of chronic liver fibrosis. A more detailed classification of patients may be needed in a further study. Fourth, as previous studies have shown that diagnostic performance of clinical biomarkers may be influenced by ASL level and necroinflammation activity, the nomogram integrating FIB-4 may add some error to the results. So, a further study with more details should be performed.

In conclusion, radiomics based on noncontrast MRI have potential in stratification of liver fibrosis. A combinational model integrating radiomics and clinical biomarkers is promising in improving diagnostic values compared to the radiomic model and clinical model.

## Figures and Tables

**Table 1:** Results of radiomics, clinical, and combinational models.

	Accuracy	Sensitivity	Specificity	Positive pred. value	Negative pred. value
Radiomics					
Train	0.84 (0.75–0.90)	0.87	0.76	0.90	0.71
Test	0.80 (0.65–0.91)	0.86	0.67	0.86	0.67

Clinic					
Train	0.85 (0.63–0.81)	0.96	0.52	0.64	0.93
Test	0.68 (0.51–0.89)	0.90	0.48	0.62	0.83

Combined					
Train	0.85 (0.76–0.91)	0.95	0.68	0.83	0.90
Test	0.76 (0.60–0.88)	0.91	0.56	0.72	0.83

## Data Availability

The data used to support the findings of this study are currently under embargo while the research findings are commercialized and are available from the corresponding author upon request (6 months) after the publication of this article.
